# Efficacy and Safety of Glecaprevir/Pibrentasvir in HCV Patients With Previous Direct-Acting Antiviral Therapy Failures: A Meta-Analysis

**DOI:** 10.3389/fmed.2020.592472

**Published:** 2020-12-03

**Authors:** Chao Shen, Haozhi Fan, Zhijun Ge, Weihua Cai, Jianguo Shao, Chen Dong, Hong Xue, Zuqiang Fu, Jun Li, Yun Zhang, Ming Yue

**Affiliations:** ^1^Key Laboratory of Infectious Diseases, Department of Epidemiology and Biostatistics, School of Public Health, Nanjing Medical University, Nanjing, China; ^2^Department of Information, First Affiliated Hospital of Nanjing Medical University, Nanjing, China; ^3^Department of Critical Care Medicine, The Affiliated Yixing Hospital of Jiangsu University, Yixing, China; ^4^Department of General Surgery, Third Affiliated Hospital of Nantong University, Nantong, China; ^5^Department of Digestive Medicine, Third Affiliated Hospital of Nantong University, Nantong, China; ^6^Department of Epidemiology and Statistics, School of Public Health, Medical College of Soochow University, Suzhou, China; ^7^Department of Severe Infectious Diseases, Third Affiliated Hospital of Nantong University, Nantong, China; ^8^Department of Infectious Diseases, First Affiliated Hospital of Nanjing Medical University, Nanjing, China; ^9^Institute of Epidemiology and Microbiology, Eastern Theater Command Centers for Disease Prevention and Control, Nanjing, China

**Keywords:** pibrentasvir, retreatment, DAAs therapy failures, meta-analysis, glecaprevir

## Abstract

**Background:** Since a greater number of hepatitis C virus (HCV) patients have access to direct-acting antiviral (DAA) based therapies, the number of patients not properly responding to prior DAA regimens is increasing. The objective of this comprehensive analysis was to assess the efficacy and safety of glecaprevir/pibrentasvir (GLE/PIB) in HCV patients who experienced previous DAA therapy failures.

**Methods:** Bibliographic databases were systematically searched for relevant articles published by November 2020. The main endpoints were sustained viral response after 12 weeks (SVR12), adverse events (AEs; any grade) and severe adverse events (SAEs). Publication bias assessment was performed using funnel plots and the Egger's test.

**Results:** Fourteen studies consisting of a total of 1,294 subjects were included in this study and the pooled estimate of SVR12, AEs and SAEs rates were 96.8% (95%CI: 95.1–98.2), 47.1% (95%CI: 26.0–69.3), and 1.8% (95%CI: 0.7–3.4), respectively. Subgroup analysis showed that pooled SVR12 rates were 97.9% (95%CI: 96.7–98.9) for Japan and 91.1% (95%CI: 87.3–94.3) for the United States; 95.8% (95%CI: 93.9–97.4) for genotype (GT)1 and 100.0% (95%CI: 99.6–100.0) for GT2; 95.3% (95%CI: 92.4–97.2) for cirrhosis and 96.3% (95%CI: 94.2–97.7) for non-cirrhosis cases. There was no publication bias included this study.

**Conclusion:** This comprehensive analysis revealed that GLE/PIB is an effective and secure retreatment option for patients who did not optimally respond to DAA treatment, especially the Asian population with GT1-2.

## Introduction

Hepatitis C virus infection is a common disease affecting ~180 million individuals worldwide (1, 2). According to the World Health Organization (WHO), ~71 million people develop chronic HCV infections that may lead to cirrhosis, hepatocellular carcinoma (HCC) and liver-related deaths (3, 4). In addition to prevention, effective regimens are critical to achieve the WHO goal of eliminating HCV as a major global public health threat by 2030 (5).

Treatment of HCV has evolved over the past decade. Before direct-acting antiviral, interferon (IFN)-based regimens were the main method of HCV, but the cure rate using this regimen was only 40–65% (6). In addition, the incidence of SAEs and discontinuation of these treatments were both frequent (7). Compared with IFN-based regimens, the efficacy and safety of DAA-based therapies for HCV resulted in dramatical improvements, including high sustained virologic response (SVR) rates, shorter treatment duration, better tolerability, and less SAEs. Despite excellent efficacy of DAA-based regimens, about 5% of patients still failed to achieve SVR and have just drawn public attention in recent years (8). Given the size of the HCV infected population, the absolute number of patients with DAA treatment failure is substantial and increasing as more patients have access to DAA-based therapies. Thus, effective and alternative treatment strategies for these individuals are particularly important.

In 2017, the combination of glecaprevir (GLE; a second-generation NS3/4 protease inhibitor) and pibrentasvir (PIB; a second-generation NS5A inhibitor) was approved and this combination shows high anti-HCV activity across genotypes 1–6 with a high *in vitro* barrier to resistance (9, 10). In clinical trials, GLE/PIB regimens showed high efficacy and favorable safety for all six major HCV genotypes (11, 12). In addition, GLE/PIB treatments were also effective and well-tolerated in patients with compensated cirrhosis or those with severe renal impairment (13, 14). Furthermore, recent studies uncovered that GLE/PIB regimens are highly effective in patients who failed to achieve SVR after prior DAA therapies (15, 16). However, the relevant researches were just conducted recently and there haven't been a lot of researches in this population. The European Association for the Study of the Liver (EASL) recommended the GLE/PIB regimen to treat treatment-experienced (pegylated IFN-a and ribavirin, pegylated IFN-a, ribavirin, and sofosbuvir, or sofosbuvir and ribavirin) HCV patients, but did not explicitly recommended this regimen to retreat patients with DAA treatment failure on account of insufficient supporting evidences.

Even though the latest guidelines from China recommended using GLE/PIB to retreat patients with prior DAA failure (17), further research is needed to increase the confidence of this recommendation. The aim of this systematic review was to assess the efficacy and safety of GLE/PIB regimens for patients who experienced DAA treatment failure.

## Method

### Search Strategy

Preferred reporting items for systematic review and meta-analyses (PRISMA) were followed to conduct this study (18). Two investigators independently performed a systematic and comprehensive literature search using multiple databases including PubMed, Embase, Web of Science, Cochrane Library, CNKI, and WanFang Data. Key search terms included (Hepacivirus OR Hepaciviruses OR Hepatitis C-Like Viruses OR Hepatitis C Like Viruses OR Hepatitis C virus OR Hepatitis C viruses OR HCV) AND (glecaprevir OR ABT-493) AND (pibrentasvir OR ABT-530). A manual search was also performed by checking related references and reviewing citations included in the selected publications. There were no language restrictions. The literature search was last updated in November 2020.

### Selection Criteria

Studies were be included if they met all the following criteria: (1) HCV patients with previous DAA therapy failures (defined as failure to achieve SVR12 after DAA treatment); (2) retreatment with GLE/PIB; (3) the primary endpoint was SVR12.

Studies were excluded if they met any of the following criteria: (1) patients without a DAA treatment history; (2) patients with a DAA treatment history, but unclear information as to whether they experienced DAA therapy failure; (3) patients who were liver transplant recipients with recurrent hepatitis C; (4) a sample size <10; (5) reports that did not provide the primary endpoint (SVR12); (6) case reports, letters, meta-analysis, editorials or reviews; (7) pharmacokinetics or pharmacodynamics studies.

### Outcome Measures

The primary outcome was the percentage of SVR12, which was defined as plasma HCV RNA below the lower limit of quantification (LLOQ) 12 weeks after end of treatment (EOT). Additional secondary primary outcomes included the percentage of patients with on-treatment breakthrough and post-treatment relapse. Breakthrough was defined as HCV RNA becoming detectable after HCV RNA below LLOQ during the treatment period. Relapse was defined as undetectable HCV RNA at EOT but became detectable within 12 weeks after. We assessed safety in terms of the incidence and intensity of AEs (any grade), common AEs (CAEs), SAEs, and discontinuation due to AEs. Analyses of secondary primary and safety outcomes included only studies reporting these data.

### Study Selection and Data Extraction

Study selection and data extraction performed by two independent researchers (CS and HZF). Study selection followed the predetermined selection criteria. Records found through primary search were initially reviewed by title and abstract. The full texts of potentially eligible studies were reviewed and eligible studies were included.

Required data were extracted from eligible studies and respected the original description. The extracted data included study characteristics (the first author's name, year of publication, region, study design, setting, publication type, sample size, subgroup number of patients, regimen, and treatment duration); patient characteristics (age, sex, HCV genotype, treatment history, resistance-associated substitutions, and presence of cirrhosis) and study outcomes (SVR12, on-treatment breakthrough, post-treatment relapse, AEs, CAEs, SAEs, and discontinuation due to AEs).

During this process, any conflicts arising between the two reviewers were resolved by consensus with the help of a third researcher (ZQF).

### Quality Assessment

The quality of studies included was assessed using the Newcastle-Ottawa quality assessment scale (NOS) for observational studies, including eight items with a total score of nine. Low quality was scored as 0–5 points, moderate quality as 6–7 points, and high quality as 8–9 points (19). The quality of randomized studies was assessed using the Cochrane Collaboration's tool. The Cochrane Collaboration's tool addresses seven specific domains including randomization, allocation concealment, blinding of subjects, blinding of outcome assessors, reporting of incomplete outcome data, selective outcome reporting, and other potential sources of bias. In each domain, every study took one of three categories: “low risk,” “high risk,” or “unclear risk” for bias (20). The quality of each included study was independently assessed by two investigators.

### Statistical Analysis

Effect sizes were collected as pooled event incidences with corresponding 95% confidence intervals (95% CI) using the inverse variance method. Zero events were estimated using Freeman-Tukey double arcsine transformation. Heterogeneity between studies was assessed using Cochran Q-statistics and *I*^2^ statistics. An *I*^2^ <50% indicated little or no heterogeneity and then the fixed-effects model was used; When the *I*^2^ ≥ 50%, this indicated moderate or substantial heterogeneity and the random-effects model was used. To effectively evaluate the efficacy and safety of GLE/PIB, we conducted subgroup analyses of SVR12 by region, setting, duration of treatment, HCV genotype, treatment history, and presence of cirrhosis. Publication bias was explored using funnel plots and the Egger's test. All statistical tests were two-sided, with a *p* < 0.05 considered as statistically significant. All statistical analyses were conducted using R version 3.6.3.

## Results

### Study Selection and Basic Information

**Figure 1:**
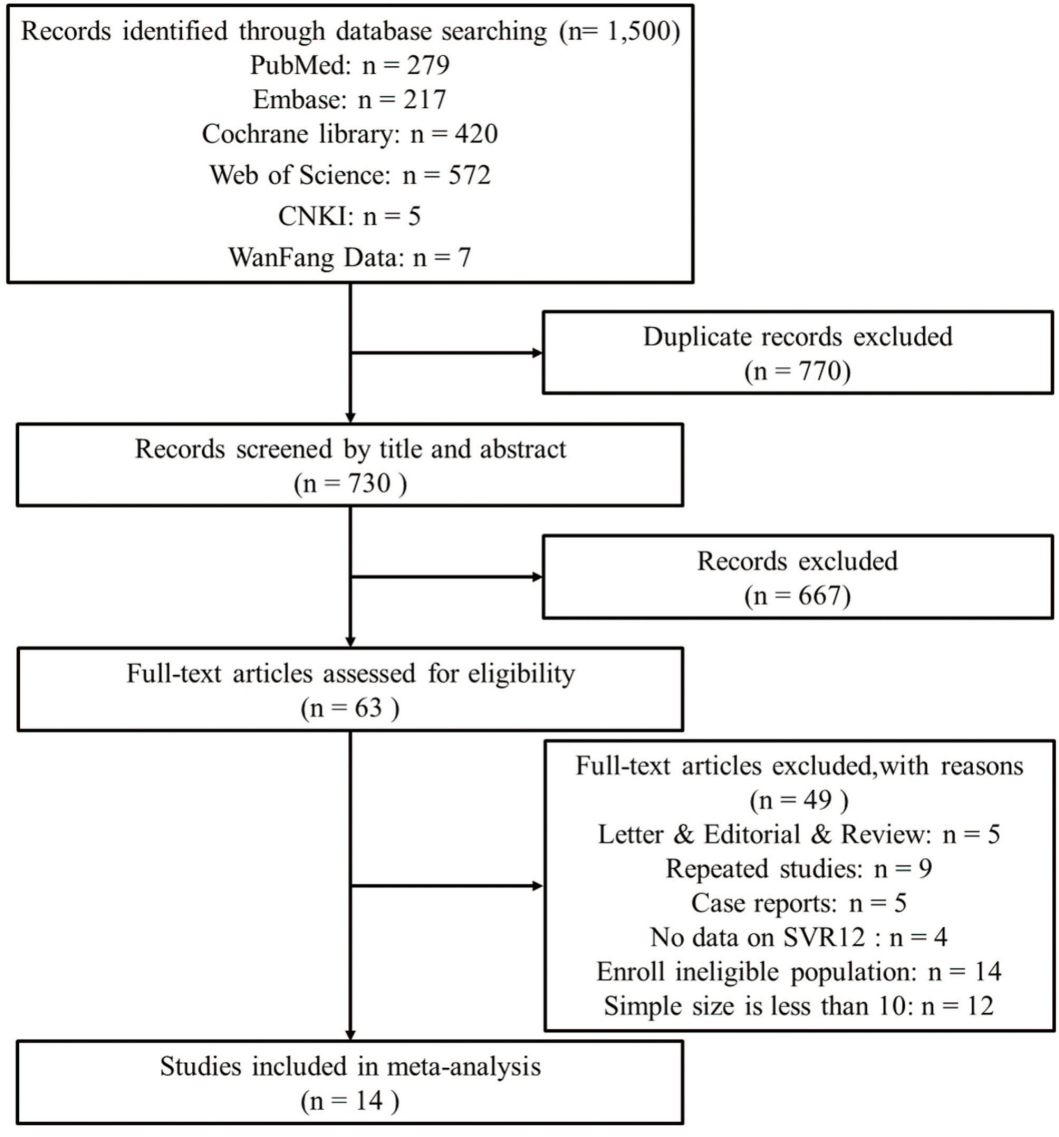
The flow diagram of literature screening and following the preferred reporting items of systematic reviews and meta-analyses (PRISMA).

Our initial search retrieved 1,500 records. After removing 770 overlapping studies, the titles and abstracts of 730 articles were screened. After assessing the full text of 63 articles, 49 articles were excluded for various reasons, and 14 articles (21–34) were eventually added to this study (Figure 1).

The 14 studies were all published since 2018, including 12 full-articles and two conference abstracts. All studies came from two regions: 12 from Japan and two from the United States. Overall 1,294 subjects showed chronic HCV infection with GT1-3, DAA treatment experience and some were presented with cirrhosis. The treatment regimens were fixed-dose combinations of GLE (300 mg/d) and PIB (120 mg/d) with or without ribavirin (RBV). Treatment courses included 12 and 16 weeks, respectively. Details of study and patient characteristics are shown in Tables 1, 2.

**Table 1:** Characteristics of the studies included in this comprehensive analysis.

**Study**	**Year**	**Study design**	**Publication type**	**Region**	**Setting**	**Regimen**	**Sample size**	**Duration (weeks)**
Shunji Watanabe	2019	Observational study	Full	Japan	Multi-center	GLE/PIB	13	12
Hayato Uemura	2019	Cohort study	Full	Japan	Single-center	GLE/PIB	42	12
Atsushi Suetsugu	2019	Observational study	Conference abstract	Japan	NA	GLE/PIB	13	12
Hitomi Sezak	2019	Prospective cohort study	Full	Japan	Single-center	GLE/PIB	88	12
Mitsutaka Osawa	2019	Observational study	Full	Japan	Single-center	GLE/PIB	30	12
Anna S. Lok	2019	Clinical trial	Full	United States	Multi-center	GLE/PIB ± RBV	177	12/16
Atsunori Kusakabe	2019	Observational study	Full	Japan	Multi-center	GLE/PIB	28	12
Masayuki Kurosaki	2019	Observational study	Conference abstract	Japan	Multi-center	GLE/PIB	237	12
Norio Akuta	2018	Observational study	Full	Japan	Single-center	GLE/PIB	20	12
Fred Poordad	2018	Clinical trial	Full	United States	Multi-center	GLE/PIB	91	12/16
Hidenori Toyoda	2019	Prospective cohort study	Full	Japan	Multi-center	GLE/PIB	199	12
Akihiro Tamori	2019	Observational study	Full	Japan	Multi-center	GLE/PIB	115	12
Ayumi Sugiura	2020	Cohort study	Full	Japan	Single-center	GLE/PIB	23	12
Akito Nozaki	2020	Observational study	Full	Japan	Multi-center	GLE/PIB	218	12/16

*GLE, glecaprevir; PIB, pibrentasvir; RBV, ribavirin; NA, not applicable*.

**Table 2:** Patient characteristics of the studies enrolled in this comprehensive analysis.

**Study**	**Year**	**Age (year)**	**Sex (M/F)**	**Cirrhosis (Yes/NO)**	**HCV-RNA (log^**10**^ IU/mL)**	**AST (IU/L)**	**ALT (IU/L)**	**HCV genotype**	**RASs in NS3 or NS5A**	**Treatment history**
Shunji Watanabe	2019	65 (52–81)	8/5	6/7	NA	NA	NA	GT 2, 3	NA	SOF/RBV
Hayato Uemura	2019	68 (36–86)	17/25	11/31	6.2 (4.0–7.2)	34 (14–156)	30 (11–132)	GT 1-3	38	DCV/ ASV, LDV/SOF, SOF/RBV, VEL/SOF+ RBV, OBV/ PTV/ r ± RBV
Atsushi Suetsugu	2019	NA	NA	NA	NA	NA	NA	GT 1, 2	NA	DCV/ASV, LDV/SOF, EBV/ GZR, SOF/RBV
Hitomi Sezak	2019	69 (58–76)	42/46	57/31	6.6 (6.0–7.0)	34 (25–55)	29 (21–56)	GT 1-3	NA	Others
Mitsutaka Osawa	2019	75 (48–86)	16/14	19/11	6.3 (5.4–7.4)	39 (18–115)	31 (9–130)	GT 1-3	26	DCV/ ASV, LDV/SOF, Other
Anna S. Lok	2019	NA	NA	50/127	NA	NA	NA	GT 1	70	SOF/LDV, SOF/VEL, SOF/DCV
Atsunori Kusakabe	2019	68.1 ± 12.5	20/8	20/8	6.06 ± 1.04	NA	59.1 ± 60.0	GT 2	NA	SOF/RBV
Masayuki Kurosaki	2019	NA	NA	NA	NA	NA	NA	GT 1	NA	Others
Norio Akuta	2018	74 (49–84)	8/12	NA	6.8 (3.1–7.5)	48 (20–123)	46 (10–128)	GT 1, 2	16	DCV/ASV, EBR/GZR, SOF/RBV, LDV/SOF, DCV/ASV
Fred Poordad	2018	NA	NA	NA	NA	NA	NA	GT 1, 4	NA	SOF/LDV, SOF/SIM, OBV/PTV/r, Others
Hidenori Toyoda	2019	69 (64–77)	90/109	93/106	6.3 (5.9–6.8)	35 (26–50)	30 (21–49)	GT 1, 2, 3	NA	Others
Akihiro Tamori	2019	NA	NA	37/78	NA	NA	NA	GT 1, 2	111	ASV/DCV, SOF/RBV, SOF/LDV, PTV/ r/OBV GZR/EBR
Ayumi Sugiura	2020	68	9/14	20/3	NA	33 (19–90)	27 (15–141)	GT 1, 2	NA	DCV/ASV, LDV/SOF, EBR/GZR, OMV/PTV/r
Akito Nozaki	2020	NA	NA	NA	NA	NA	NA	GT 1, 2, 3	NA	Others

*AST, aspartate aminotransferase; ALT, alanine aminotransferase; GT, genotype; RASs, resistance-associated substitutions; SOF, sofosbuvir; RBV, ribavirin; DCV, daclatasvir; ASV, asunaprevir; LDV, ledipasvir; VEL, velpatasvir; OBV, ombitasvir; PTV, paritaprevir; r, ritonavir; EBV, elbasvir; GZR, grazoprevir; SIM, simeprevir; EBR, elbasvir; Others, any other combination of DAA regimens; NA, not applicable*.

### Quality of the Included Studies

Twelve observational studies were assessed by NOS. Among these studies, three were of high quality, six were of moderate quality and the others were of low quality. The quality assessment scores are shown in Supplementary Table 1.

Two clinical trials (26, 32) were assessed using the Cochrane Collaboration's tool. Among these assessed items, randomization, allocation concealment, reporting of incomplete outcome data, selective outcome reporting, and other potential sources of bias were reported in these two different studies. These results are represented in Supplementary Figure 1.

### Efficacy of Outcomes

#### SVR12

**Figure 2:**
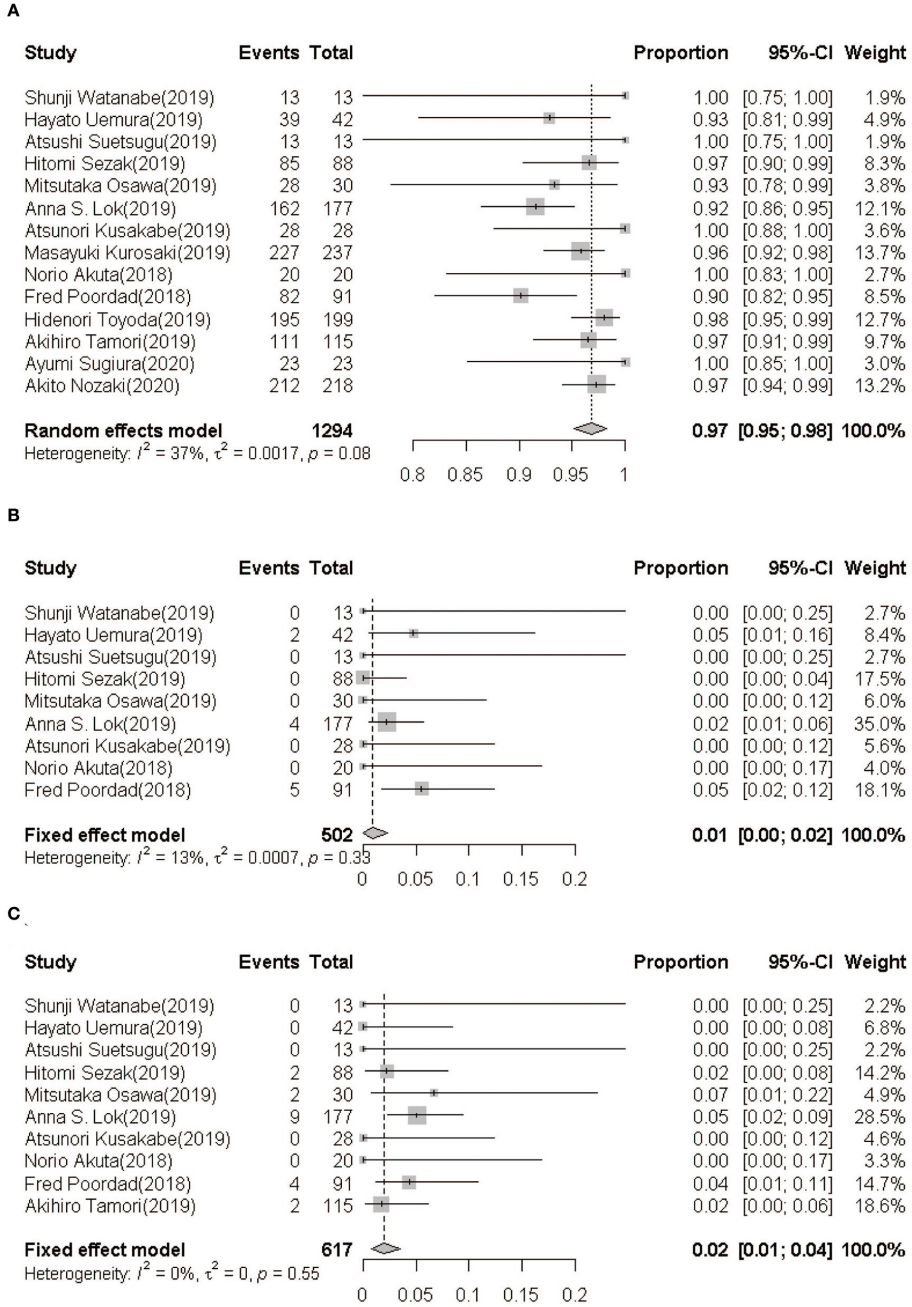
**(A)** SVR12 rate following GLE/PIB treatment. **(B)** On-treatment breakthrough rate following GLE/PIB treatment. **(C)** Post-treatment relapse rate following GLE/PIB treatment. GLE/PIB, glecaprevir/pibrentasvir; CI, confidence interval.

Data on SVR12 rates of GLE/PIB retreatment for HCV infection were available in all studies (1,294 cases). The pooled estimation of the SVR12 rate from the random-effect model was 96.8% (95%CI: 95.1–98.2, *I*^2^ = 37.1%, *P* = 0.08) (Figure 2A).

#### Breakthroughs and Relapses

In nine studies (21–27, 29, 32), only 11 of the 503 HCV patients retreated with GLE/PIB showed an on-treatment breakthrough with a pooled rate being 0.92% (95%CI: 0.08–2.32, *I*^2^ = 13.1%, *P* = 0.33) (Figure 2B). Furthermore, 19 of the 617 patients from 10 studies (21–27, 29, 31, 32) showed a post-treatment relapse with a pooled rate being 1.96% (95%CI: 0.78–3.50, *I*^2^ = 0.0%, *P* = 0.55) (Figure 2C).

### Subgroup Analysis of the SVR12 Rate

Based on settings, regions, genotypes, treatment history, treatment durations and the presence or absence of cirrhosis, we conducted subgroup analyses as detailed in Table 3. The rate of SVR12 was 96.0% (95%CI: 93.4–97.5) in multi-center and 96.1% (95%CI: 92.3–98.0) in single-center studies. Approximately 97.9% of patients (95%CI: 96.7–98.9) in Japan presented achieved SVR12 rate, while the SVR12 rate of the United States subgroup was 91.1% (95%CI: 87.3–94.3). As for genotypes, the pooled SVR12 rates in GT1, GT2, and GT3 were 95.8% (95%CI: 93.9–97.4), 100.0% (95%CI: 99.6–100.0), and 100.0% (95%CI:66.74–100.0). In subgenotype subgroups, GT1a, GT1b, GT2a, and GT2b were 90.3% (95%CI: 84.4–94.2), 94.8% (95%CI: 90.3–97.3), 100.0% (95%CI: 99.7–100.0), and 100.0% (95%CI: 98.3–100.0), respectively. Among the patients who had treatment history available for analysis, the SVR12 rates of sofosbuvir/ribavirin (SOF/RBV), daclatasvir/asunaprevir (DCV/ASV), ledipasvir/sofosbuvir (LDV/SOF), ombitasvir/paritaprevir/ritonavir (OBV/PTV/r), elbasvir/grazoprevir (EBV/GZR), and other DAAs were 100.0% (95%CI: 99.2–100.0), 97.6% (95%CI: 92.9–100.0), 99.3% (95%CI: 86.0–100.0), 100.0% (95%CI: 73.9–100.0), 97.4% (95%CI: 62.0–100.0), and 96.5% (95%CI: 95.1–97.7), respectively. Nine studies including 784 patients provided data for subgroup analysis with the presence or absence of cirrhosis. The SVR12 rates for patients with or without cirrhosis were 95.3% (95% CI: 92.4–97.2) and 96.3% (95% CI: 94.2–97.7), respectively.

**Table 3:** SVR12 by settings, regions, genotypes, treatment history, treatment durations, and the presence or absence of cirrhosis.

**Response**	**SVR12 (*****N*** **=** **1,294)**		**Heterogeneity**		P^**^-value	**Studies**
	**Total, n/N**	**Rate (95%CI)**	***I^**2**^* (%)**	P^*^		***N***
Overall	1238/1294	96.8 (95.1–98.2)	37.1	0.08		14
By settings					0.9483	
Single-center	195/203	96.1 (92.3–98.0)	0.0	0.91		5
Multi-center	1030/1078	96.0 (93.4–97.5)	56.9	0.03		8
By regions						
Japan	994/1026	97.9 (96.7–98.9)	0.0	0.77	0.0003	12
United States	244/268	91.1 (87.3–94.3)	0.0	0.67		2
By genotypes					0.0064^a^	
1	637/674	95.8 (93.9–97.4)	0.0	0.46	0.1325^b^	9
1a	131/145	90.3 (84.4–94.2)	0.0	1.00		3
1b	164/173	94.8 (90.3–97.3)	0.0	0.80		5
2	165/165	100.0 (99.6–100.0)	0.0	1.00	0.7802^c^	9
2a	72/72	100.0 (99.7–100.0)	0.0	0.98		7
2b	53/53	100.0 (98.3–100.0)	0.0	0.99		7
3	6/6	100.0 (66.7–100.0)	0.0	0.99		4
By treatment history					0.2787	
SOF/RBV	81/81	100.0 (99.2–100.0)	0.0	0.99		7
DCV/ASV	120/126	97.6 (92.9–100.0)	0.0	0.81		5
LDV/ SOF	27/29	99.3 (86.0–100.0)	0.0	0.95		5
OBV/PTV/r	7/7	100.0 (73.9–100.0)	0.0	0.94		3
EBV/GZR	8/9	97.4 (62.0–100.0)	0.0	0.70		4
Others	829/863	96.5 (95.1–97.7)	47.6	0.09		6
By treatment durations					0.2349	
12	1121/1169	95.9 (94.6–96.9)	43.6	0.15		14
16	117/125	93.6 (87.7–96.8)	0.0	0.46		2
By the presence or absence of cirrhosis					0.4681	
Cirrhosis	304/319	95.3 (92.4–97.2)	0.0	0.63		9
Non-cirrhosis	448/465	96.3 (94.2–97.7)	39.0	0.28		9

*SOF, sofosbuvir; RBV, ribavirin; DCV, daclatasvir; ASV, asunaprevir; LDV, ledipasvir; OBV, ombitasvir; PTV, paritaprevir; r, ritonavir; EBV, Elbasvir; GZR, grazoprevir r; Others, any other combination of DAA regimen; CI, confidence interval*.

* *Test of heterogeneity*.

** *Test for subgroup differences*.

a *Test for subgroup (genotype1-3) difference*.

b *Test for subgroup (genotype1a and 1b) difference*.

c *Test for subgroup (genotype2a and 2b) difference*.

Additionally, the SVR12 rate of the Japan subgroup was higher than the United States subgroup (*P* = 0.0003) and the GT1 was lower than the GT2-3 (*P* = 0.0064). However, there were no significant differences between the other subgroups analyzed.

### Safety

Six studies reported numbers for AEs, CAEs, SAEs, and discontinuation due to AEs were 288, 273, 12, and 4, respectively. The pooled rates of AEs, CAEs, SAEs, and discontinuation due to AEs were 47.1% (95%CI: 26.0–69.3), 45.2% (95%CI: 25.3–66.7), 1.8% (95%CI: 0.7–3.4), and 0.1% (95%CI: 0.0–0.9), respectively (Table 4). The main CAEs were fatigue (6.8%), headache (8.1%), nausea (4.1%), pruritus (11.8%), and appetite loss (1.3%). Furthermore, three studies observed treatment-related laboratory abnormalities in seven patients, including elevation of total bilirubin (5/254) and serum ALT levels (2/254).

**Table 4:** Rate of safety outcomes GLE/PIB for patients with HCV.

**Outcomes**	**Safety**		**Heterogeneity**	**studies**	
	**Total, n/N**	**Rate%(95%CI)**	***I^**2**^* (%)**	***P***	
AEs	288/550	47.1 (26.0–69.3)	95.1	<0.01	6
CAEs	273/550	45.2 (25.3–66.7)	94.8	<0.01	6
SAEs	12/550	1.8 (0.7–3.4)	0.0	0.47	6
Discontinuation due to AEs	4/550	0.1 (0.0–0.9)	0.0	0.70	6

*GLE/PIB, glecaprevir/pibrentasvir; CI, confidence interval; AEs, any adverse events; CAEs, common adverse events; SAEs, serious adverse events*.

### Publication Bias and Sensitivity Analysis

Funnel plots for the SVR12 rate are shown in Supplementary Figures 2, 3. The Egger's test for evaluating publication bias showed that no publication bias was identified in these studies (*t* = 1.72, *P* = 0.11). Furthermore, the results from the sensitivity analysis manifested that the pooled estimate of SVR12 did not depend on a single study (Supplementary Figure 4).

## Discussion

This study provided estimates regarding the efficacy and safety following GLE/PIB retreatment for patients who experienced prior DAA treatment failure. These results indicated that GLE/PIB for patients experiencing DAA therapy failure can achieve high SVR12 rates at 12 and 16 weeks, regardless of sex, age, genotype, the presence or absence of cirrhosis or other demographic factors. The rates of SAEs and discontinuation due to adverse events were minimal in GLE/PIB. Thus, GLE/PIB is an effective and secure retreatment option for patients who experience DAA treatment failure and this is critical information for global HCV treatment guidelines.

In this meta-analysis, the pooled SVR12, breakthrough, and relapse rates were 96.8% (95%CI: 95.1–98.2), 0.92% (95%CI: 0.08–2.32), and 1.96% (95%CI: 0.78–3.50), respectively. Compared with sofosbuvir/velpatasvir/voxilaprevir (35), and sofosbuvir/elbasvir/grazoprevirs ± ribavirin (36) retreatments, their SVR12 rates were similar. The incidence of failure of GLE/PIB was lower than other regimens, such as sofosbuvir/daclatasvir (5.7%) and sofosbuvir/velpatasvir (3.4%) (37). Although sofosbuvir/velpatasvir/ voxilaprevir was considered as a highly effective option for the re-treatment of HCV patients, the AEs (100%), SAEs (6.5%), and discontinuation due to AEs rates (5.2%) rates were higher than what was observed for GLE/PIB (AEs = 47.1%, SAEs = 1.8%, and discontinuation due to AEs = 0.1%) (35, 38), which explained an advantage for the GLE/PIB regimen.

Our findings revealed that the SVR12 rate among individuals in Japan was significantly higher than individuals in the United States. Only two studies (*n* = 268) were derived from the United States, so this analysis may be restricted by a finite sample size. Alternatively, a possible explanation was that therapy efficacy was related to race. Kanwal et al. reported differences among gender and race subgroups in the DAA treatment group (39). Most patients in Japan were Asians while most patients in the United States were White, Hispanic or Black. In addition, retreatment data from Asians were limited and available research showed that the SVR24 rate was 91.2% in patients with previous therapy failures (40). Therefore, our study suggested that GLE/PIB was of great significance for the retreatment of Asians with HCV therapy failures and more studies are needed for further evaluation in the United States.

In terms of genotype, there were significant differences among GTs 1-3 while no significant differences were in subgenotype subgroups (GT1a vs. GT1b; GT2a vs. GT2b). In previous studies, patients with GT3 infection showed lower SVR rates compared with other GTs. However, our data were inconsistent with previous studies since the SVR12 rates of GTs2-3 were 100% higher than GT1 (95.4%). On one hand, the sample size of GT3 was small (*n* = 6) which caused poor accuracy and reliability so more GT3 cases should be included to obtain enough evidence. We inferred that GLE/PIB still had a high efficacy for GT3 in patients with previous DAA therapy failures, considering that a systematic review demonstrated that GLE/PIB had distinct performance (SVR12 rate = 96.1%) when it came to the treatment of GT3 (41). On the other hand, five studies have found baseline resistance-associated substitutions (RASs) in NS3 or NS5A region and these RASs were mostly in subjects with GT1 (261/384). Moreover, at least 25 of 37 patients with GT1 who failed to achieve SVR12 had detected RASs in the NS3 or NS5A region. RASs were produced by the error-prone replication of HCV that could decrease efficacy of the DAA regimens (42). We suspected that the existence of RASs caused the decrease of the SVR12 rate in GT1. Even so, the SVR12 rate was still ≥ 95% in GT1, which suggested an ideal curative effect. Thus, GLE/PIB, one of the NS3/4/NS5A combination regimens, is extremely effective and a strong choice for the HCV population with RASs.

Currently, there are three major classes of antiviral HCV drugs including: inhibitors of the NS3/NS4A protease (PIs), inhibitors of the NS5A complex and inhibitors of the NS5B polymerase (43). In our study, main DAA treatment histories included SOF/RBV and DCV/ASV, belonging to the three classes of DAAs mentioned. As the first pan-genotypic DAA agent that was approved, SOF was widely used in many countries, but about 10% of people treated with SOF-containing regimens did not achieve SVR (44). DCV is a DAA agent that was approved by the European Medicines Agency for combination with other medicinal products for treating chronic HCV genotype 1, 3, or 4 infections (45). Administered with an NS3 protease inhibitor (ASV), DCV achieves greater than a 90% HCV eradication rate, while around 5–10% will not be cured (46). Furthermore, except for four studies without a clear DAA treatment history, these prior DAA treatments mostly were PIs and NS5A or NS5B inhibitor-containing regimens. Although DAA treatment histories were varied, GLE/PIB obtained favorable SVR rates (>95%), especially sofosbuvir-containing regimens (100%), which implied its wide application and fantastic efficacy.

Interestingly, treatment duration did not increase the response rate of GLE/PIB in our subgroup analysis. However, only two studies contained a 16-weeks GLE/PIB therapy period and showed that the SVR rates of the 16-weeks treatment subgroup were higher than the 12-weeks treatment subgroup. These two studies came from the United States with relatively low SVR12 rates. This may explain why the 16-weeks treatment SVR rate did not increase. In addition, there was no significant observed difference between patients with or without cirrhosis. It can be inferred that re-treatment with 12 weeks of GLE/PIB is highly effective in HCV patients with or without cirrhosis and future guidelines should consider recommending a 12-weeks therapy.

Our comprehensive analysis exhibited several strengths. First, our study was the first to evaluate the efficacy and safety of GLE/PIB for HCV patients with previous DAA therapy failures. We screened 14 studies including 1,294 individuals, which allowed us to accurately assess the pooled SVR12 rate, breakthrough, relapse, AE and SAE rates of populations who had previous DAA treatment failure. In addition, the heterogeneity among the included studies for most analyses was small, which indicated that this study is reliable and may help clinicians effectively retreat HCV subjects.

However, despite these strengths, this study still contained several limitations. First, only the efficacy and safety rates were analyzed along with the 95% CI. The relative risk (RR) for the various subgroups was not analyzed due to the absence of a control group. Second, most included studies were from Japan and focused on patients with GT1-3. Our data may not be relatable to other nations and genotypes. Third, some studies offered the frequency of prior DAA treatment but we did not perform subgroup analysis on this subject due to insufficient data.

## Conclusion

This comprehensive analysis supports that the GLE/PIB regimen has strong efficacy and increased safety for HCV patients with previous DAAs therapy failures, especially the Asian population with GT1-2 regardless of treatment duration, and the presence or absence of cirrhosis. Furthermore, GLE/PIB is appropriate for subjects with various DAA treatment failures, such as sofosbuvir-containing regimens.

## Data Availability Statement

The original contributions presented in the study are included in the article/Supplementary Materials, further inquiries can be directed to the corresponding author/s.

## Author Contributions

CS, MY, and HF participated in the design of the study. CS, HF, ZG, WC, and ZF took charge of literature retrieval, data collection, and quality control. CS, JS, and JL performed the statistical analysis. HX, HF, CD, and MY contributed to analysis. CS, HF, and WC wrote the paper. All authors read and approved the final manuscript.

## Conflict of Interest

The authors declare that the research was conducted in the absence of any commercial or financial relationships that could be construed as a potential conflict of interest.
